# Platelets and VEGF blood levels in cancer patients

**DOI:** 10.1038/sj.bjc.6690673

**Published:** 1999-09

**Authors:** E Gunsilius, G Gastl

**Affiliations:** Division of Hematology and Oncology, University Hospital, Anichstr. 35, Innsbruck, 6020, Austria


the rationale for the potential efficacy of finasteride in preventing prostate cancer. We began our study fully expecting to observe beneficial effects on prostate cancer. Unfortunately we did not.

Although, we, too, were somewhat surprised by the low incidence of prostate cancer in the observational arm at the 1-year follow-up biopsy, we doubt that Coltman would have found acceptable a trial utilizing possibly inappropriate historical controls. The notion that you can change an observation in a trial and modify statistical values is hardly novel, and belies the entire purpose of statistical testing. Coltman raises the issue that if the cancer detection rate had been 7/27 and 1/25 in the treated and untreated groups, the *P*-value of the difference would be 0.051. Although this does not reach 'statistical significance', we would have considered such a difference to be very worrisome.

Coltman then states that if the patients with PIN are removed, the difference in cancer detection rates is meaningless. Two points can be made: (1) a clear and important conclusion of our study is that the presence of PIN is a strong risk factor for developing a positive biopsy after finasteride treatment; (2) in men without PIN the effect of finasteride on the diagnosis of prostate cancer is 'meaningless' because of small numbers. However, the much larger study referred to by Coltman of McConnel et al (1998) did not find a protective effect of finasteride either. In the latter trial, a total of 3040 men were randomized to receive finasteride or placebo for the treament of symptoms of benign prostatic hyperplasia. Treatment was for 4 years; in order to monitor for prostate cancer, 645 men underwent prostate biopsies during the study (325 men in the finasteride group and 320 men in the placebo group). The incidence of prostate cancer was 5% in each group, indicating no reduction in prostate cancer risk in a large population of lower risk men undergoing long-term treatment with finasteride.

Coltman repeatedly points to the small size of our trail. However, we were faced with the serious dilemma in the interim analysis of our study, that we might be causing harm. We decided that we could not justify continuing to accrue additional subjects.

Our trial, while small, remarkably provides the only data of any kind to date on the biological effects of finasteride on human prostate tissue. We strongly feel that studies such as ours are essential before embarking on large scale, long-term multi-institutional trials in healthy individuals. We undertook our study because of the dearth of any such relevant information.

As Chairman of the Southwest Oncology Group under whose auspices the US National Prostate Cancer Prevention Trial is being conducted, we believe the results of our study merit Dr Coltman's attention, and should not be dismissed on dubious statistical grounds.

*RK Ross, MC Pike, E Skinner and RJ Cote*
*University of Southern California, School of Medicine,*
*1441 Eartlake Avenue, Los Angeles, CA 90033-0800, USA*

### REFERENCES

Cote RJ, Skinner EC, Salem CE, Mertes SJ, Stanczyk FZ, Henderson BE, Pike MC and Ross RK (1998) The effect of finasteride on the prostate gland in men with elevated serum prostate-specific antigen levels. *Br J Cancer* **78**: 413–418

McConnel JD, Bruskewitz R, Walsh P, Andriole G, Lieber M, Holtgrewe LH, Albertson P, Roehbom CG, Nickel JC, Wang DZ, Taylor AM and Waldstrelcher J (1998) The effect of finasteride on the risk of acute urinary retention and the need for surgical treatment among men with benign prostatic hyperplasia. *New Engl J Med* **338**: 557–563

Ross RK, Bernstein L, Lobo RA, Shimizu H, Stanczyk F, Pike MC and Henderson BE (1992) 5-alpha reductase activity among Japanese and US white and black males. *Lancet* **339**: 887–890

### Platelets and VEGF blood levels in cancer patients

### Sir

It was with great interest that we read the letter to the Editor from Vermeulen et al (1999) regarding the accuracy of the measurement of vascular endothelial growth factor (VEGF) serum levels (VEGF-SL) and VEGF plasma levels (VEGF-PL) in cancer patients (Vermeulen et al, 1999). We have measured about 1000 serum samples for VEGF and should thus like to comment on their observations based on our findings:

1. We agree with Vermeulen et al that interindividual and intra-individual fluctuations of serum VEGF levels can, at least in part, be explained by variations of blood platelet counts. Another factor that may contribute to the variability of serum VEGF levels is the platelet volume. This notion is substantiated by our findings from patients receiving myeloablative chemotherapy. In these patients, screening of VEGF-SL was begun prior to the platelet nadir ($< 20$ G $l^{-1}$) and continued until the platelet counts had been recovered. VEGF was measured in 140 serum samples and 54 corresponding plasma samples by enzyme-linked immunosorbent assay (ELISA), essentially as described by Vermeulen et al. In line with their results, we found a striking correlation between peripheral blood platelet counts and absolute values of VEGF-SL ($r = 0.8$; $P < 0.001$), but not with VEGF-PL. Like Vermeulen and colleagues, we noted a broad inter-individual and intra-individual variability of VEGF-SL values. Thus, we hypothesized that VEGF-SL may not only depend on blood plaletet counts but also on the plaletet size. Platelets freshly released from the bone marrow following myeloablative chemotherapy may differ in size from those produced during a steady-state of myelopoiesis. To test this hypothesis, we compared the mean platelet volumes (MPV) prior to the platelet nadir with the MPV during the time of platelet reconstitution by using an electronic particle counter. In peripheral blood, the MPV before the platelet nadir indeed proved to be significantly lower than afterwards. Even if VEGF-SL values were corrected for the actual blood platelet count ($VEGF^{PLT}$ = VEGF-SL $\times 10^6$ platelets/actual blood platelet count), $VEGF^{PLT}$ values prior to the platelet nadir were significantly lower than those measured afterwards (Figure 1).
2. In vivo, platelet activation or platelet destruction causes major increments in VEGF blood levels: we detected high VEGF levels in both serum and plasma samples indicating a massive release of VEGF during intravasal platelet destruction in a patient with thrombotic-thrombocytopenic purpura.
3. Tumour cells and/or blood platelets may be the major sources of VEGF in cancer patients, particularly in metastatic disease. In fact, the majority of cancer patients with metastatic disease show elevated VEGF levels in serum (Kraft et al, 1999). Moreover, increasing VEGF serum levels may herald tumour relapse or progression. In a patient with breast cancer undergoing adjuvant high-dose chemotherapy, we noted a rapid

© 1999 Cancer Research Campaign

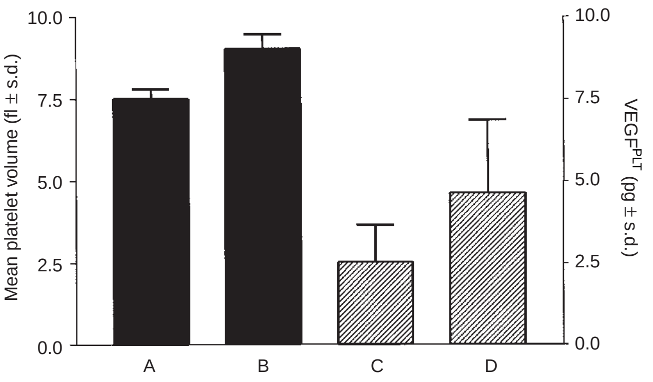

**Figure 1** Mean platelet volumes (MPV, black columns) and corresponding $VEGF^{PLT}$ (hatched columns) in a patient undergoing myeloablative chemotherapy. MPV before the platelet nadir (A) were lower than those thereafter (B, $P = 0.001$). Also, the $VEGF^{PLT}$ are lower before the platelet nadir (C) than thereafter (D). $VEGF^{PLT}$ correlated to the MPV ($r = 0.59$)

increase in the $VEGF^{PLT}$ value from 2.1 ± 1.0 pg during the time of remission to 6.75 pg at the time of clinical relapse. This increment in $VEGF^{PLT}$ could be due to a release of VEGF from a fast-growing tumour mass and/or from tumour-activated platelets (Pinedo et al, 1998). We did indeed find tumour cells to induce platelet activation and release of VEGF from activated platelets (manuscript in preparation).

In summary, we agree with Vermeulen and colleagues that VEGF levels in serum may be a useful tumour marker that relates to the disease stage, tumour progression and tumour-induced platelet activation. To correctly interpret serum VEGF levels, however, blood platelet counts and the platelet size must be taken into consideration.

*E Gunsilius and G Gastl*
*Division of Hematology and Oncology, University Hospital, Anichstr. 35, 6020 Innsbruck, Austria*

### REFERENCES

Vermeulen PB, Salven P, Benoy I, Gasparini G and Dirix LY (1999) Blood platelets and serum VEGF in cancer patients [letter]. *Br J Cancer* **79**: 370–373
Kraft A, Weindel K, Ochs A, Marth C, Zmija J, Schumacher P, Unger C, Marme D and Gastl G (1999) Vascular endothelial growth factor in the sera and effusions of patients with malignant and nonmalignant disease. *Cancer* **85**: 178–187
Pinedo HM, Verheul HM, D'Amato RJ and Folkman J (1998) Involvement of platelets in tumour angiogenesis? *Lancet* **352**: 1775–1777

### Effect of acetic acid on telomerase activity in premalignant and malignant cervical lesions

### Sir

We read with great interest the report by Mutirangura et al (1998) on defining a correlation between telomerase activity and human papillomavirus (HPV) in normal control tissue and in benign, premalignant and malignant cervical lesions.

We are convinced of the conclusion that there may be two roles of telomerase in the cervix (the first one would present in benign lesions; the second is associated with cancer development and activated during the late stage of multistep carcinogenesis). However, we have doubt about the low percentages of telomerase activity reported for high-grade squamous intraepithelial lesions (SILs) as 40%, and probably for others, especially after reading the article by Changchien et al (1998). The studies of many authors revealed that 25–58% of high-grade SILs exhibited telomerase activity (Kyo et al, 1997; Pao et al, 1997; Zheng et al, 1997). However, Changchien et al reported a relative high percentage of telomerase activity (77.1%) in high-grade SILs. The large discrepancy between the results of previous studies and the results of them were explained by the methods of cervical tissue collection. They claimed that they increased the detection rate of telomerase activity by making the tissues submitted for telomerase assay free of acetic acid.

It is well known that swabbing of cervix with 3–5% acetic acid is a crucial step when colposcopy is performed. Since some of the samples of Mutirangura et al were also obtained by this way, those samples could have been affected by acetic acid. Due to the reason that pH of 5% acetic acid is too low and exposure time is too long, telomerase protein is probably irreversibly denatured by this process (Lodish et al, 1995).

We would like to stress that if 'acetic acid' factor is taken into consideration in such studies, much higher telomerase detection rates and eventually much more accurate results of it are possible to be obtained.

*IH Güllü and M Kurdoğlu*
*Institute of Oncology, Hacettepe University, Sihhiye 06100, Ankara, Turkey.*

### REFERENCES

Chang Chien C, Lin H, Leung SW, Hsu C and Cho C (1998) Effect of acetic acid on telomerase activity in cervical intraepithelial neoplasia. *Gynecol Oncol* **71**: 99–103
Kyo S, Takakura M, Ishikawa H, Sasagawa T, Satake S, Tateno M and Inouc M (1997) Application of telomerase assay for the screening of cervical lesions. *Cancer Res* **57**: 1863–1867
Lodish H, Baltimore D, Berk A, Zipursky Lawrence S, Matsudira P and Darnell J (1995) A protein can be unfolded by heat, extreme pH and certain chemicals in molecular cellular biology. In: *Protein Structure and Function*, 3rd edn, pp. 73–75. Scientific American Books
Mutirangura A, Sriuranpong V, Termrunggraunglert W, Tresukosol D, Lertsaguansinchai P, Voravud N and Niruthisard S (1998) Telomerase activity and human papillomavirus in malignant, premalignant and benign cervical lesions. *Br J Cancer* **78**: 933–939
Pao CC, Tseng CJ, Lin CY, Yang FP, Hor JJ, Yao DS and Hsueh S (1997) Differential expression of telomerase activity in human cervical cancer and cervical intraepithelial neoplasia lesions. *J Clin Oncol* **15**: 1932–1937
Zheng PS, Iwasawa T, Yokoyama M, Nakao Y, Pater A and Sugimori H (1997) Telomerase activation in in vitro and in vivo cervical carcinogenesis. *Gynecol Oncol* **66**: 222–226

*© 1999 Cancer Research Campaign*

